# 초임부의 자기분화, 심리적 불편감 및 부부적응이 태아애착에 미치는 영향

**DOI:** 10.4069/kjwhn.2020.11.12

**Published:** 2020-12-15

**Authors:** Bu Kyung Kim, Mi-Hae Sung

**Affiliations:** Graduate school, Inje University, Busan, Korea; 인재대학교 대학원; Institute of Health Science and College of Nursing, Inje University, Busan, Korea; 인제대학교 간호대학, 건강과학연구소

**Keywords:** Maternal-fetal relations, Pregnant women, Psychological distress, 모아애착, 임부, 심리적 불편감

## Abstract

**Purpose:**

The purpose of this study was to identify the influence of self-differentiation, psychological discomfort, and marital dyadic adjustment on maternal-fetal attachment in primigravida.

**Methods:**

In total, 108 primigravida participated in this descriptive correlational study. The participants answered self-report questionnaires. Data were collected from January to May, 2020, and were analyzed using descriptive statistics, the t-test, analysis of variance, Pearson correlation coefficients, and hierarchical multiple regression with SPSS for Windows ver. 23.0.

**Results:**

The mean age of the primigravida was 31.66 years. The mean score for the degree of maternal-fetal attachment was 76.81 out of 96 points. Participants’ scores for maternal-fetal attachment differed significantly based on age (t=2.08 *p*=.039) and marital status (t=2.05, *p*=.043). Maternal-fetal attachment was significantly negatively correlated with psychological discomfort (r=–.39, *p*<.001), and significantly positively correlated with self-differentiation (r=.36, *p*<.001) and marital dyadic adjustment (r=.36, *p*<.001). Self-differentiation explained 24.1% of variance in participants’ maternal-fetal attachment, and its effect was statistically significant (F=7.79, *p*<.001).

**Conclusion:**

In primigravida, more self-differentiation was associated with stronger maternal-fetal attachment. To strengthen maternal-fetal attachment in primigravida, educational program that increases the level of self-differentiation and minimizes their psychological discomfort may be helpful for first time pregnant women. Additionally, it is recommended to provide nursing interventions to encourage couples to work together throughout the gestational period.

## Introduction

부모가 되는 것은 성인기의 사회화 과정 중 한 단계이며, 임신은 의미 있는 생활 사건으로 초임부는 임신한 사실을 확인한 순간부터 태아의 존재를 인식하게 되고, 어머니-태아 관계가 발달하게 된다[1]. 임신을 통해 임부는 태아에게 정서적 유대감과 감정을 갖게 되고, 태동을 느끼면서 감정이 더욱 강해지며 태아에게 적극적으로 반응한다[2]. 이러한 심리적 결속을 통해 태아와 상호작용 또는 애정을 나타내는 행위에 참여하는 정도를 태아애착이라 하는데[3], 태아애착은 임부의 발달 과제이며, 출산 전 어머니와 아이의 관계의 출발점이다[4]. 태아가 태내에 있어 임부가 직접 눈으로 확인할 수 없지만, 태아의 초음파 사진이나 태동 등을 통해 태아의 존재감을 느끼며, 이를 통해 태아애착을 발달시키게 된다[5,6]. 임부와 태아가 나눈 안정적인 교감을 통해 형성된 강한 태아애착은 출생 이후 자녀와의 긍정적인 상호작용을 예측하는 주요 요인이다[2]. 임신기의 태아애착은 출산 후 자녀와의 애착관계와 자녀의 신체적, 심리적 발달에도 지속적인 영향을 미치며[7], 태아애착 정도가 낮을 경우 태아에 대해 부정적인 생각과 감정을 초래한다[2]. 특히 초임부는 처음으로 부모 됨을 준비하는 단계이기 때문에 초임부의 안정적인 태아애착 형성은 임부와 태아에게 매우 중요하다[1,7].

자기분화(self-differentiation)는 Bowen [8]의 가족체계이론의 초석을 이루는 개념으로 ‘한 개인이 어머니와의 정서적 융합에서 서서히 벗어나 자신의 정서적 자주성을 확립하는 과정’이다. 자기분화가 되었다는 것은 주변의 영향과 자신의 감정에 휘둘리지 않는 성숙한 인격체로서의 독립된 자기 자신으로 존재한다는 의미이며, 자기분화 수준이 높은 사람은 감정과 이성이 균형을 이루고 자기 생각과 느낌을 유지하며, 타인의 가치와 신념을 있는 그대로 존중한다[9]. 자기분화 수준은 우울, 불안 등의 심리적 안녕 및 가족 내의 여러 문제와 연관이 있으며[8], 경임부와 초임부를 포함한 임부를 대상으로 한 연구에서 자기분화 수준이 높을수록 태아애착 정도가 높은 것으로 나타났다[2]. 초임부는 원 가족의 자식의 역할에서 부모 역할로의 전환점에 있기 때문에 초임부의 정서적 자주성 확립이 중요하며, 초임부의 자기분화 정도가 태아와의 애착관계에 영향을 미칠 것이라 예측되어 이에 대해 확인할 필요가 있다.

초임부는 일생에 중요한 변화인 임신으로 인해 부모가 된다는 설렘과 행복감을 느끼는 동시에 스트레스로 인한 소화불량, 두통과 같은 신체적 증상, 불안, 우울 등과 같은 심리적 불편감을 경험한다[10]. 임신기에 경험하는 심리적 불편감은 태아에 대한 애착도 감소하게 하며[2], 출산 후 자녀에게 부정적인 영향을 주고[11], 자녀와의 정서적 상호작용인 애착관계에도 영향을 준다[12]. 이와 같이 심리적 불편감은 초임부의 태아애착에도 영향을 미칠 것이라 예측되어 이에 관한 규명이 필요할 것으로 보인다.

배우자와의 관계를 나타내는 부부적응(marital dyadic adjustment)은 결혼생활에서 발생하는 여러 가지 갈등을 대화를 통해 해결하고 결혼생활의 안정과 만족을 추구하는 부부의 정서적 또는 행동적 과정이며[13], 부부간의 적응은 임부의 심리적 상태에 매우 중요한 영향을 미친다[14]. 부부가 서로 잘 적응하지 못하고 배우자의 지지가 약할수록 임부는 부정적 정서를 경험하며[15], 원만한 부부적응이 실현되고 결혼생활에 만족이 높을수록 태아애착 행위가 높다고 하였다[16]. 따라서, 부부적응은 임부의 심리적 및 정서적 상태에 영향을 미치고 초임부의 태아애착 행위에 영향을 주는 중요한 요인이 될 수 있다. 이와 같이, 자기분화와 심리적 불편감 및 부부적응이 태아 애착에 영향을 미칠 변수로 예측되며, 이들이 초임부의 태아 애착에도 영향을 줄 것인지 알아볼 필요가 있다. 초임부를 대상으로 한 태아애착에 관한 연구로, 체험중심 산전 프로그램의 효과[17], 예비 아버지의 애착기술훈련이 초임부의 태아애착에 미치는 효과[18], 초임부와 유산력이 있는 임부의 태아애착 행위 비교[19] 등이 있지만, 국·내외적으로 초임부를 대상으로 자기분화, 심리적 불편감, 부부적응 및 태아애착을 다룬 연구는 없는 실정이다. 초임부가 첫 부모가 되기 위한 준비과정으로 태아애착 정도를 높여 분만과 육아 과정까지 잘해나갈 수 있도록 돕기 위해 초임부의 태아애착에 미치는 영향요인을 확인하는 것이 중요하다[17]. 이에 연구자는 초임부를 대상으로 자기분화, 심리적 불편감 및 부부적응이 태아애착에 영향을 미치는 요인을 파악함으로써, 초임부의 태아애착 증진을 위한 간호 중재 전략에 필요한 기초자료를 제공하고자 한다.

구체적인 목적은 다음과 같다.

· 대상자의 자기분화, 심리적 불편감, 부부적응 및 태아애착 정도를 파악한다.

· 대상자의 일반적 특성 및 산과적 특성에 따른 태아애착의 차이를 파악한다.

· 대상자의 자기분화, 심리적 불편감, 부부적응 및 태아애착 간의 상관관계를 파악한다.

· 대상자의 태아애착에 영향을 미치는 요인을 파악한다.

## Methods

Ethics statement: This study was approved by the Institutional Review Board of Inje University (IRB-2019-10-009-001). Informed consent was obtained from the participants.

### 연구 설계

본 연구는 초임부의 자기분화, 심리적 불편감, 부부적응 및 태아애착 정도와 이들 변수 간의 관계를 파악하고 태아애착에 미치는 영향요인을 확인하기 위한 상관성 조사연구이다.

### 연구 대상

본 연구는 부산시에 소재하는 2개의 여성 전문병원을 방문한 만 19세 이상의 초임부를 대상으로 하였으며, 임신합병증과 다른 질병이 있는 자는 제외하였다. 해당 병원의 산부인과에서 산전 진찰을 받기 위해 외래에서 대기 중인 대상자에게 연구자가 연구의 목적과 선정기준에 관해 설명하였다. 연구 참여를 수락한 대상자에게 연구윤리 준수사항과 언제라도 동의 철회가 가능하다는 점 등을 포함한 연구 전반에 관해 설명한 후 동의서를 받고 설문조사를 실시하였다.

본 연구의 표본은 G*Power 3.1.9.2 프로그램을 사용하여 유의수준 .05, 검정력 .85로 하고, 임부를 대상으로 자기분화, 심리적 불편감이 태아애착에 유의한 상관관계를 보인 연구[2]와 부부적응이 태아애착과 유의한 상관관계를 보인 선행연구[4]를 근거로 중간 효과크기 .15, 예측변인 5개(연령, 학력, 자기분화, 심리적 불편감, 부부적응)로 투입할 경우, 최소 표본 수가 102명이 산출되었다. 탈락률 20%를 고려하여 총 123명을 대상으로 자료 수집을 실시하였고 그 중 119부가 회수되었다(회수율 96.7%). 회수된 119부 중 응답이 불성실한 11부를 제외한 108부를 최종 분석에 활용하였다.

### 연구 도구

본 연구에 사용한 모든 도구는 사용 이전 도구 개발자 및 한국어 번안자의 허락을 받았다.

#### 자기분화

Chung과 Cho [20]가 개발한 자기분화 척도를 사용하였다. 이 도구는 총 38문항으로 정서적 반응 9문항, 자기 입장 8문항, 타인과의 융합 7문항, 정서적 단절 5문항, 정서적 융합 9문항으로 구성되어 있다. 각 문항은 6점 Likert 척도로 ‘전혀 그렇지 않다’ 0점에서 ‘매우 그렇다’ 5점이고 점수 범위는 0–190점까지이며, 부정문항은 역코딩하여 점수가 높을수록 자기분화 정도가 높은 것을 의미한다. 자기분화 척도의 개발 당시 도구[20]의 신뢰도(Cronbach’s α)는 .89였고, 본 연구에서는 .91이었다.

#### 심리적 불편감

간이 정신진단검사(Brief Symptoms Inventory-18) [21]를 국내에서 사용 가능한 한국판 척도로 번안한 도구[22]를 사용하였다. 이 도구는 총 18문항으로 신체화 6문항, 우울 6문항, 불안 6문항으로 구성되어 있다. 각 문항은 5점 Likert 척도로, ‘전혀 없다’ 0점에서 ‘매우 심하다’ 4점까지로 점수 범위는 0–72점까지이며, 점수가 높을수록 심리적 불편감 정도가 높은 것을 의미한다. 개발 당시 도구[21]의 신뢰도(Cronbach’s α)는 신체화 .74, 우울 .84, 불안 .79였고, 번안한 도구[22]는 .89였으며, 본 연구에서는 .93이었다.

#### 부부적응

Spanier [13]가 개발하고 Busby 등[23]이 보완, 개정한 부부적응 도구(Revised Dyadic Adjustment Scale)를 번안한 한국형 개정판 부부적응 도구[24]를 사용하였으며, 이 도구는 총 14개 문항으로 일치도 6문항, 만족도 4문항, 응집도 4문항으로 구성되어 있다. 각 문항은 6점 Likert 척도로, 각 문항은 ‘항상 그렇지 않다’ 1점에서 ‘항상 그렇다’ 6점까지로 점수 범위는 14–84점까지이며, 부정문항은 역코딩하여 점수가 높을수록 부부적응 정도가 높은 것을 의미한다. 개발 당시 도구[13]의 신뢰도(Cronbach’s α)는 .93이었고, 번안한 도구[24]는 .87–.91이었으며, 본 연구에서는 .86이었다.

#### 태아애착

태아애착도구(Maternal-Fetal Attachment Scale) [3]를 번안한 도구[25]를 사용하였다. 이 도구는 총 24개 문항으로 자신과 태아의 구별 3문항, 태아와의 상호작용 5문항, 역할 수용 4문항, 태아의 특성과 의도에 대한 추측 6문항, 자기 헌신 6문항으로 구성되어 있다. 각 문항은 4점 Likert 척도로, ‘전혀 안 했다’ 1점에서 ‘항상 그랬다’ 4점으로 점수 범위는 24–96점이며, 점수가 높을수록 태아애착 정도가 높음을 의미한다. 개발 당시 도구[3]의 신뢰도(Cronbach's α)는 .85였고, 번안한 도구[25]는 .89였으며, 본 연구에서는 .90이었다.

#### 자료 수집

본 연구는 2020년 1월 14일부터 5월 4일까지 자료 수집을 하였다. 해당 병원의 기관장과 부서장에게 본 연구의 목적과 취지, 연구윤리 준수사항 등을 포함한 내용을 설명한 후 해당 기관에서 연구수행을 허락받고 진행하였다. 해당 병원의 산전 진찰을 위해 대기 중인 임부 중 외래 간호사가 선정기준에 맞는 대상자를 연구자에게 알려준 후 연구자가 개별적으로 접촉하여 대상자에게 직접 연구의 목적을 밝히고, 자발적인 연구 참여 의사를 확인 후 참여 의사를 밝힌 대상자에게만 언제든지 동의 철회가 가능하다는 것과 자료의 비밀 유지에 관한 내용 등을 설명한 후 동의서를 받고 설문조사를 실시하였다. 연구자가 서류 봉투에 담긴 설문지를 배부하였으며, 완성된 설문지는 익명으로 서류 봉투에 밀봉하여 회수하였다. 설문지 작성 시간은 약 10분 정도 소요되었으며, 연구에 참여한 모든 대상자에게 소정의 선물을 제공하였다.

### 자료 분석

수집된 자료는 IBM SPSS Win ver. 24.0 (IBM Corp., Armonk, NY, USA)을 이용하여 분석하였다. 대상자의 일반적 특성 및 산과적 특성과 자기분화, 심리적 불편감, 부부적응 및 태아애착 정도는 기술통계로 분석하였으며, 일반적 특성과 산과적 특성에 따른 태아애착의 차이는 t-test와 one-way ANOVA, 사후검정은 Scheffé로 분석하였다. 자기분화, 심리적 불편감, 부부적응 및 태아애착 간의 관계는 Pearson correlation coefficient로 분석하였고, 태아애착 영향요인은 위계적 다중 회귀 분석(hierarchical multiple regression)으로 분석하였다.

## Results

### 대상자의 일반적 특성과 산과적 특성

대상자의 연령은 평균 31.66세이고 31세 이상인 군이 56.5%로 많았으며, 결혼기간은 평균 25.51개월이고 24개월 이상인 군이 43.5%로 많았다. 대상자의 결혼상태는 기혼인 군이 94.4%로 많았고, 학력은 대졸 이상인 군이 95.4%로 많았다. 대상자의 직업은 있는 경우가 64.8%였고, 월수입은 평균 514.03만 원으로 401만 원 이상인 군이 59.3%로 많았다. 대상자의 종교는 없는 경우가 63.9%였고, 동거가족은 남편인 경우가 87%로 많았다. 대상자의 임신기간은 평균 24.72주로 임신 3기인 군이 46.3%였고, 임신 계획 여부는 ‘예’로 응답한 군이 73.1%였다(Table 1).

### 대상자의 자기분화, 심리적 불편감, 부부적응 및 태아애착 정도

대상자의 자기분화 점수는 총 190점 만점에 평균 105.64점, 표준편차 21.52점이었고, 심리적 불편감 점수는 총 72점 만점에 평균 10.59점, 표준편차 10.36점이었다. 대상자의 부부적응 점수는 총 84점 만점에 평균 66.61점, 표준편차 8.90점이었으며, 태아애착 점수는 총 96점 만점에 평균 76.81점, 표준편차 10.36점이었다(Table 2).

### 대상자의 일반적 특성과 산과적 특성에 따른 태아애착

대상자의 태아애착에 차이를 보이는 특성은 연령(t=2.08 *p*=.039)과 결혼상태(t=2.05, *p*=.043)로, 30세 이하인 군이 31세 이상인 군보다 태아애착이 높았으며, 기혼인 경우가 미혼인 경우보다 태아애착이 높았다(Table 1).

### 대상자의 자기분화, 심리적 불편감, 부부적응 및 태아애착의 상관관계

대상자의 태아애착은 자기분화(r=.36, *p*<.001)와 정적 상관관계가, 심리적 불편감(r=–.39, *p*<.001)과 부적 상관관계가 있었고, 부부적응(r=.36, *p*<.001)과는 정적 상관관계가 있었다. 자기분화는 심리적 불편감(r=–.42, *p*<.001)과 부적 상관관계가 있었고, 부부적응(r=.28, *p*=.003)과는 정적 상관관계가 있었다. 심리적 불편감은 부부적응(r=–.42, *p*<.001)과 부적 상관관계가 있었다(Table 3).

### 대상자의 태아애착에 영향을 미치는 요인

대상자의 태아애착에 영향을 미치는 요인을 알아보기 위하여 위계적 다중 회귀분석을 실시하였다. 우선, 모형 1에서는 일반적 특성 중 태아애착에 유의한 차이를 나타낸 연령과 결혼상태를 투입하였고, 모형 2에서는 자아분화, 심리적 불편감, 부부적응을 추가로 투입하였다. 이 가운데 명목변수인 연령(30세 이하 기준)과 결혼상태(미혼 기준)는 가변수(dummy variable)로 처리하여 분석하였다. 정규 p-p 도표 기울기가 45도로 나타나 선형성이 확인되었고, Kolmogorov-Smirnov 검정 결과 z=0.071 (*p*>.05)로 정규성이 확인되었다. 분석 전 오차항들 간 자기 상관이 있는지 알아보기 위하여 Durbin-Watson 통계량을 구한 결과 2.01로 2 근방으로 나타나 오차항들 간 자기상관은 없었다. 독립변수들 간 다중공선성이 있는지 공차한계와 variance inflation factor (VIF)를 구한 결과 공차한계는 .71–.97, VIF는 1.02–1.39로 나타나 독립변수들 간 다중공선성은 없었다. 대상자의 일반적 특성을 투입한 모형 1의 경우, 연령이 30세 이하일 경우(t=–2.18, *p*=.031)와 결혼상태가 기혼일 경우(t=2.15, *p*=.033)가 태아애착에 영향을 미치며, 모형 1의 설명력은 6.3%로 분석되었다(F=4.57, *p*=.012). 연령이 30세 이하일수록(β=–.20, *p*=.031) 결혼상태가 기혼일수록(β=.20, *p*=.033) 태아애착에 영향을 미치는 것으로 나타났다. 모형 2에서 자기분화, 심리적 불편감, 부부적응을 추가로 투입한 결과 설명력이 19.6% 증가하여(F=9.21, *p*<.001), 24.1%로 분석되었다(F=7.79, *p*<.001). 개별 변인인 심리적 불편감(t=–1.95, *p*=.054)과 부부적응(t=1.98, *p*=.050)은 유의하지 않았으며, 자기분화 정도(t=2.21, *p*=.029)는 유의하였고, 자기분화 정도가 높을수록(β=.21, *p*=.029) 태아애착에 영향을 미치는 것으로 나타났다(Table 4).

## Discussion

본 연구는 초임부의 자기분화, 심리적 불편감, 부부적응 및 태아애착 정도와 이들 변수 간의 관계를 확인하고 태아애착에 미치는 영향요인을 파악하기 위한 것으로 본 연구 결과를 토대로 논의하고자 한다.

대상자의 태아애착 점수는 총 96점 만점에 평균 76.81이었는데, 이는 같은 도구를 사용한 초임부의 태아애착을 측정한 연구[17]에서 평균 73.9점으로 나온 결과와 유사하며, 같은 도구를 사용하여 초임부를 대상으로 한 연구가 없어 직접적인 비교는 어렵지만 정상임부를 대상으로 한 연구[6]의 평균 69.4점보다 높은 것이다. 이러한 결과는 정상임부를 대상으로 한 연구[4]에 경임부가 포함되어 있으므로 초임부가 경임부보다 태아애착 점수가 높게 나타난 결과[4,14,16]와 같은 맥락으로 생각된다. 이러한 연구결과는 본 연구에서 대상자가 출산 경험이 없는 초임부이기 때문에 경임부보다 태아에 대한 신비감, 기대감이 높아 더 많은 애정과 관심으로 이어지면서 대상자의 태아애착 정도가 높게 나온 것으로 생각한다[4].

대상자의 일반적 특성 및 산과적 특성에 따른 태아애착은 연령과 결혼상태에서 통계적으로 유의한 차이가 있었다. 이는 정상임부를 대상으로 한 연구[4]에서 태아애착은 연령에 따른 차이를 보이나 교육, 직업, 종교, 수입, 동거가족, 임신 계획 유무에 따른 차이는 없었던 결과와 부분적으로 일치한다. 본 연구에서 통계적으로 유의하게 연령에 따른 차이가 있었으며, 나이가 적을수록 태아애착 평균 점수가 높았다. 그리고 법적인 결혼상태가 기혼인 경우가 미혼인 경우보다 태아애착 정도가 높았는데, 법적인 결혼상태가 가족 간의 결속력 생성과 초임부의 정서적 안정에 도움을 주는 요소로 작용하여 태아애착에 영향을 미쳤을 것이라 생각된다. 그러나, 연령과 법적인 결혼상태 외에는 태아애착에 차이를 보인 일반적 특성 및 산과적 특성은 없었기 때문에 본 연구에서 다룬 변수 외에 다양한 일반적 특성 및 산과적 특성을 포함하여 초임부의 태아애착 차이를 비교한 반복 연구가 필요할 것이다.

대상자의 태아애착은 자기분화와 정적 상관관계를, 심리적 불편감과 부적 상관관계를, 부부적응과 정적 상관관계를 나타냈다. 이는 임신 후반기 부부를 대상으로 한 연구[2]에서 태아애착이 자기분화의 하위요인과 정적 상관관계를, 심리적 불편감의 하위요인과 부적 상관관계를 나타낸 것이나, 고위험 임부를 대상으로 한 연구[26]에서 태아애착이 부부적응과 정적 상관관계를 나타낸 것과 부분적으로 일치하는 것이다. 이러한 결과로 볼 때, 초임부의 자기분화 정도를 높이고 심리적 불편감을 최소화하며 부부적응 정도를 강화하도록 격려한다면 초임부의 태아애착을 증진 시킬 수 있을 것으로 생각된다. 따라서, 초임부의 자기분화 정도를 높여주며 심리적 불편감은 최소화하고 부부가 함께 첫 임신이라는 사건을 기쁘게 받아들여 조화롭게 적응해 나갈 수 있도록 가족이 함께 참여하는 태아애착 증진 프로그램을 개발할 필요가 있다.

대상자의 태아애착에 영향을 미치는 요인은 자기분화였다. 이는 임신 후반기 부부를 대상으로 한 연구[2]에서 태아애착에 미치는 영향으로 자기분화, 심리적 불편감 순으로 나타난 결과와 일치하지만, 본 연구의 회귀분석 모형에서는 심리적 불편감이 유의한 변수로 나타나지 않았다. 높은 수준의 자기분화는 태아에 대해 긍정적인 애착을 발달시키므로[2] 임신이라는 사건에서 임부의 내·외적 변화에 따른 감정적 동요를 차단하고 자기 신념에 따라 스스로 의사결정을 내리며 산전관리를 이행할 수 있도록 돕는 교육 프로그램이 필요하다고 생각된다.

태아애착에 영향을 미치는 영향요인으로 자기분화 정도는 총 190점 만점에 평균 105.64점이었는데, 같은 도구를 사용하여 초임부를 대상으로 한 연구가 없어 직접적인 비교는 어렵지만, 임신 후반기 부부를 대상으로 한 연구[2]에서 임부의 점수가 평균 111.79점으로 나온 결과와 유사하다. Bowen [8]의 제안에 따라 자기분화 수준을 백분위 점수로 환산하여 0–25점, 25–50점, 50–75점, 75–100점 등 범주별로 나타내고 각 범주에 속한 사람의 분화수준을 특징할 수 있다. 본 연구의 초임부의 자기분화 점수는 55.6점이었는데 이는 분화척도에서 중간(50–70점 사이)의 범주에 드는 사람으로, 본 연구에 참여한 초임부들은 충분히 자기 스스로 몇 개의 결정을 내리며, 본질적인 문제에 대해 잘 정의된 신념과 의견을 가지고 있고, 심한 스트레스를 겪게 되면 상당한 정서적, 신체적, 사회적 징후를 발달시킬 수 있으나 회복도 빠른 특징을 가진다고 할 수 있다. 원 가족의 자식 역할에서 부모가 되는 전환점에 있는 초임부의 자기분화 정도는 중요하며, 남편 또한 임부와 함께 부모가 되는 전환점에서 정신적 성숙도는 중요하므로 초임 부부를 대상으로 자기분화 정도를 파악할 필요성이 있다.

심리적 불편감과 부부적응은 태아애착에 영향을 미치는 요인으로 나타나지는 않았으며, 본 연구에서 대상자의 심리적 불편감 정도는 총 72점 만점에 평균 10.59점이었는데, 같은 도구를 사용하여 초임부를 대상으로 한 연구가 없어 직접적인 비교는 어렵지만 임신 후반기 부부를 대상으로 한 연구[2]에서 평균 28.62점으로 나온 결과보다 낮은 것이다. 이러한 결과는 본 연구에서 임신 기간에 제한을 두지 않아 후기 임부의 심리적 불편감과 정확한 차이를 비교할 수 없지만, 선행연구에서 임신 후기의 여성은 분만일에 대한 부담감으로 불안을 더 많이 호소한다[17]. 심리적 불편감의 하위요인에 불안이 포함되어 있으므로, 본 연구의 초임부의 평균 임신기간이 24.72주인 것과 관련되어 심리적 불편감이 임신 후기 여성보다 낮게 나타난 것으로 생각된다. 대상자의 부부적응 정도는 총 84점 만점에 평균 66.61점이었는데, 이는 같은 도구를 사용한 고위험 임부를 대상으로 한 연구[26]에서 평균 64.56점으로 나온 결과와 유사하고, 정상임부를 대상으로 한 연구[27]에서 평균 59.62점으로 나온 결과보다 높은 것이다. 임신은 부부가 함께 겪어가는 과정임을 감안할 때 처음 임신을 경험하는 초임부와 태아가 위험해 처해질 가능성을 가지고 있는 고위험 임부의 부부 결속력이 높아 정상임부보다 부부적응 정도가 높게 나타난 것으로 생각된다.

본 연구에서 심리적 불편감과 부부적응은 태아애착과 유의한 상관관계였으나, 태아애착에 미치는 영향요인으로는 나타나지 않았기 때문에 심리적 불편감과 부부적응이 태아애착에 미치는 영향에 대해서 반복연구가 필요하며, 그 외에 다른 변수를 포함하여 초임부의 태아애착에 대한 다각적인 실증적 연구가 계속 이루어져야 할 것이다.

본 연구는 국내에서 처음으로 초임부를 대상으로 자기분화, 심리적 불편감, 부부적응 및 태아애착 간의 관계를 파악하고 태아애착의 영향 요인을 분석하여 초임부의 태아애착을 높이기 위한 중재 전략 수립에 필요한 기초자료를 마련하였다는 데 그 의의가 있다. 특히 자기분화는 주로 가족치료에서 다루어졌지만, 개인적으로 건강한 삶과 건강한 부모 역할에도 기여하며 자녀의 양육과 자녀 성장에도 영향을 미치므로, 부모 역할의 시작점인 초임부를 대상으로 자기분화 정도를 확인해 보았다는 의의가 있다.

본 연구는 일부 지역 2개 병원의 초임부를 대상으로 편의 표집하였으므로 일반화에 신중을 기하여야 한다는 제한점이 있다. 또한 초임부를 대상으로 자기분화의 개념을 적용한 연구가 없어 결과의 비교가 어려웠으므로 이를 토대로 초임부의 자기분화 정도를 높일 수 있는 간호중재를 개발하며, 임부가 굳건하고 독립된 자아로서 부모가 되기 위한 준비과정을 돕는 태아애착 증진 프로그램을 개발 및 적용하여 효과를 검증하는 추후 연구를 제언한다.

## Figures and Tables

**Table 1.** Differences in maternal-fetal attachment according to general and obstetric characteristics (N=108)

Characteristic	Categories	n (%)	Mean±SD	t/F	*p*
Age (year)	≤30	47 (43.5)	79.14±9.00	2.08	.039
	≥31	61 (56.5)	75.01±11.04		
Length of marriage (month)	≤11	31 (28.7)	76.16±11.49	0.08	.919
	12–23	30 (27.8)	77.05±9.81		
	≥24	47 (43.5)	77.09±10.18		
Marital status	Married	102 (94.4)	77.30±10.31	2.05	.043
	Unmarried	6 (5.6)	68.50±7.81		
Level of education	≤High school	5 (4.6)	71.80±16.30	–1.10	.270
	≥Bachelor’s degree	103 (95.4)	77.05±10.05		
Occupation	Yes	70 (64.8)	77.50±9.38	0.93	.354
	No	38 (35.2)	75.55±12.00		
Monthly family income (10,000 Korean won)	≤300	24 (22.2)	74.16±9.51	1.77	.174
	301–400	20 (18.5)	75.10±12.42		
	≥401	64 (59.3)	78.34±9.84		
Religion	Yes	39 (36.1)	77.69±10.23	0.66	.511
	No	69 (63.9)	76.31±10.48		
Living together	Spouse	94 (87.0)	77.03±10.33	0.39	.677
	Spouse and other family	9 (8.3)	76.77±11.56		
	Other family	5 (4.6)	72.80±10.20		
Trimester	First	20 (18.5)	75.70±9.42	0.48	.231
	Second	38 (35.2)	75.00±11.27		
	Third	50 (46.3)	78.64±9.88		
Planned pregnancy	Yes	79 (73.1)	77.21±9.85	0.66	.510
	No	29 (26.9)	75.72±11.76		

**Table 2.** Degree of self-differentiation, psychological discomfort, marital dyadic adjustment, and maternal-fetal attachment (N=108)

Variable	Mean±SD	Minimum	Maximum	Possible range
Self-differentiation	105.64±21.52	62	150	0–190
Psychological discomfort	10.59±10.36	0	57	0–72
Marital dyadic adjustment	66.61±8.90	36	82	14–84
Maternal-fetal attachment	76.81±10.36	48	96	24–96

**Table 3.** Correlations among self-differentiation, psychological discomfort, marital dyadic adjustment, and maternal-fetal attachment (N=108)

Variable	r (*p*)			
	Self-differentiation	Psychological discomfort	Marital dyadic adjustment
Psychological discomfort	–.42 (<.001)	1	
Marital dyadic adjustment	.28 (.003)	–.42 (<.001)	1
Maternal-fetal attachment	.36 (<.001)	–.39 (<.001)	.36 (<.001)

**Table 4.** Factors affecting maternal-fetal attachment (N=108)

Variable	Model 1			Model 2		
	B	β	t (*p*)	B	β	t (*p*)
Age^†^	–4.26	–.20	–2.18 (.031)	–3.48	–.16	–1.96 (.052)
Marital status^†^	9.09	.20	2.15 (.033)	5.06	.11	1.30 (.196)
Self-differentiation				0.10	.21	2.21 (.029)
Psychological discomfort				–0.19	–.19	–1.95 (.054)
Marital dyadic adjustment										0.21	.18	1.98 (.050)
Adjusted R^2^	.06			.24		
F (df), *p*	4.57 (2), .012			7.79 (5), <.001		
R^2^change (F, *p*)				.19 (9.21, <.001)		

† The dummy variable references were age (≤ 30 years) and marital status (unmarried).
